# First description of *Escherichia coli* producing CTX-M-15- extended spectrum beta lactamase (ESBL) in out-patients from south eastern Nigeria

**DOI:** 10.1186/1476-0711-11-19

**Published:** 2012-07-23

**Authors:** Ifeanyichukwu R Iroha, Charles O Esimone, Sandra Neumann, Lennart Marlinghaus, Miriam Korte, Florian Szabados, Sören Gatermann, Martin Kaase

**Affiliations:** 1 Department of Applied Microbiology, Faculty of Biological Sciences, Ebonyi State University, P.M.B. 053, Abakaliki, Nigeria; 2 Department for Medical Microbiology, German Reference Laboratory for Multidrug-Resistant Gram- negative bacteria, Ruhr- University Bochum, Bochum, Germany; 3 Department of Pharmaceutical Microbiology and Biotechnology, Nnamdi Azikiwe University, P.M.B. 5025, Awka, Nigeria

**Keywords:** Outpatients, ESBL, CTX-M, *Escherichia coli*

## Abstract

We studied the presence of extended spectrum beta lactamases (ESBLs) in 44 clinical isolates of *Escherichia coli* collected from out-patients in two university teaching hospitals in South-Eastern Nigeria. Species identification was performed by standard microbiology methods and re-confirmed by MALDI-TOF technology. Phenotypic characterization of ESBL enzymes was done by double disc synergy test and presence of ESBL genes was determined by specific PCR followed by sequencing. Transfer of plasmid DNA was carried out by transformation using *E. coli* DH5 as recipient strain. Phenotypic characterization identified all isolates to be ESBL positive. 77% of strains were from urine, 13.6% from vaginal swabs and 9.0% from wound swabs. 63.6% were from female patients, 68% were from outpatients and 95.5% from patients younger than 30 years. All ESBL producers were positive in a PCR for *bla*_CTX-M-1_ cluster, in exemplary strains *bla*_CTX-M-15_ was found by sequencing. In all strains IS*Ecp1* was found upstream and ORF477 downstream of *bla*_CTX-M_. PCR for *bla*_TEM_ and *bla*_OXA-1_ was positive in 93.1% of strains, whereas *bla*_SHV_ was not detected, aac(6′)-Ib-cr was found in 97.7% of strains. RAPD analysis revealed seven different clonal groups named A through G with the majority of the strains (65.9%) belonging to clone A. Transfer of an ESBL plasmid with co-resistance to gentamicin, kanamycin, tobramycin, doxycycline and trimethropim-sulfamethoxazole was successful in 19 (43.2%) strains. This study showed a high rate of CTX-M-1 cluster - ESBLs in South-Eastern Nigeria and further confirms the worldwide spread of CTX-M ESBL in clinical isolates.

## Introduction

Microbial resistance is a growing major public health issue and a strong concern for the medical community. Production of β-lactamases is a major means by which Gram-negative bacteria exhibit resistance to β-lactam antibiotics [1]. Extended spectrum β-lactamases (ESBLs) are a group of enzymes that can hydrolyze a variety of β-lactams including cephalosporins like ceftazidime, cefotaxime, ceftriaxone and monobactams like aztreonam in addition to penicillins but do not hydrolyze cephamycins like cefoxitin. Most of the ESBLs also have the ability to hydrolyze fourth generation cephalosporins like cefepime [2]. Until recently, ESBL-producing organisms were viewed as hospital-acquired or health care-associated pathogens, i. e. affecting patients who had typically been in hospitals or other health care facilities like nursing homes [3,4]. In the last decades however, these infections have increasingly been recognized in patients who had no prior contact with the health care system [5,6]. *E. coli* strains producing CTX-M type ESBLs drive this new epidemic. Reports on this newer group of ESBLs, coined CTX-M for their preferential hydrolysis of cefotaxime over ceftazidime started to emerge in *E. coli* in the late 1990s [7]. Studies have indicated that the genes for CTX-M type ESBLs were mobilized from the chromosomes of *Kluvera* spp. to *E. coli* plasmids through transposon-mediated events [8,9]. Currently five clusters of CTX-M type ESBLs have been identified namely the CTX-M-1, CTX-M-2, CTX-M-8, CTX-M-9, and CTX-M-25 clusters. CTX-M-14 belonging to the CTX-M-9 cluster and CTX-M-15 belonging to the CTX-M-1 cluster are particularly associated with community-acquired isolates. In the present study, we investigated the molecular epidemiology of clinical isolates of ESBL producing *Escherichia coli* isolated from outpatients in South-Eastern Nigeria.

## Materials and methods

A total of 44 clinical isolates of *E. coli* were isolated from patients attending University of Nigeria teaching hospital, Enugu (HA, n = 28) and Ebonyi State University teaching hospital Abakaliki (HB, n = 16). All isolates were characterized using standard microbiology methods and re-confirmed by MALDI-TOF [10,11]. Extended spectrum beta lactamase enzymes were determined by double disc synergy test (DDST) method with disks of ceftazidime, cefotaxime, ceftriaxone, cefepime, and aztreonam (30 μg each) placed at a distance of 15 mm (center to center) from a disk containing amoxicillin plus clavulanic acid (20 and 10 μg, respectively) [12].

### Isolation of genomic DNA of ESBL *E. Coli*

Genomic DNA of ESBL *E. coli* was prepared using the NucleoSpin Kit (Macherey & Nagel, Germany) following manufacturer’s instructions.

### Detection of resistance gene using PCR method

Primers used for amplification of resistance genes are shown in Table 1. Cycling conditions were as follows: initial denaturation at 94°C for 5 min, followed by 34 cycles of denaturation at 94°C for 30 s and a final extension step at 72°C for 5 min. Annealing temperatures differed according to the primer pair used and were 42°C for TEM, 47°C for SHV, 52°C for OXA-1, 58.8°C for CTX-M-1. Amplified PCR products were separated on 0.8% agarose gels, stained with ethidium bromide and visualized under UV illumination. Appropriate positive and negative controls were used in all cases [13-15]. Some PCR products were sequenced with 3730 sequencer (applied biosystem). The nucleotide and deduced amino acid sequence were analyzed and compared to sequence available over the Internet at the National Center for Biotechnology Information (http:IIwww.ncbi.nlm.gov). A PCR with specific primers for *aac(6′)-Ib-cr* was performed [16] with the primers shown in Table 1 and an annealing temperature of 45°C and followed by restriction with *Bse*GI. The genetic environment of *bla*_CTX-M_[17] was analyzed by PCR for ISE*cp1* upstream and ORF477 downstream with the primers shown in Table 1.

**Table 1:** Primers used in this study

**Primer**	**Sequence**	**Amplicon size**	**Source**
TEM_seq	TCAACATTTCCGTGTCG	860 bp	Schlesinger *et al.*[13]
TEM_rev	CTGACAGTTACCAATGCTTA		
SHV_seq	ATGCGTTATATTCGCCTGTG	776 bp	Schlesinger *et al.*[13]
SHV_rev	AGATAAATCACCACAATGCGC		
CTX-M_seq	RGMAGYGYRMCGCTKYATGCSC	730 bp	Schlesinger *et al.*[13]
CTX-M_rev	ARTARGTSACCAGAAYVAGCGG		
OXA-1_fw	GGATAAAACCCCCAAAGGAA	369 bp	Karisik E *et al.*[15]
OXA-1_rev	TGCACCAGTTTTCCCATACA		
ISEcp1_5′	TTCAAAAAGCATAATCAAAGCC	Variable	Eckert C *et al.*[17]
MA1_rev	ACYTTACTGGTRCTGCACAT		
CTX-M-1_54 0	GCGTGATACCACTTCACCTC	460 bp	this study
orf477_rev	GAAGGAGAACCAGGAACCAC		this study
aac6-Ib-fw	TTGCGATGCTCTATGAGTGGCTA	482 bp	Park CH *et al.*[16]
aac6-Ib-rev	CTCGAATGCCTGGCGTGTTT		
RAPD_1290	GTGGATGCGA		Pacheco AB et al. [18]

### Randomly amplified polymorphic DNA (RAPD) analysis

RAPD was performed with all the 44 ESBL positive strains using a single primer as showed in Table 1 with annealing temperature of 37°C [18].

### Transformation studies

Plasmid DNA was extracted with the Qiagen Midi Kit PC100 (Qiagen, Germany). Transformation of plasmids was carried out by electroporation with *E. coli* DH5 cells as recipient strain at 2.5 kV, 25 F and 200 using a Gene–Pulser (Bio-Rad, Hemel hempstead, UK). Samples from donor and recipient strains were used as controls. Transformants growing on the selection plates (cefotaxime 2 mg/L, Sigma Aldrich Poole UK) were subjected to DDST to confirm the presence of ESBL genes and were examined for co-transfer of other antibiotic resistance determinants present in the donor clinical isolates by disk diffusion.

## Results

The overall result showed that of 44 *E. coli* strains studied 34 (77.2%) were from urine, 6 (13.6%) from vaginal swabs and 4 (9.0%) from wound swabs, 28 (63.6%) were from female patients. Remarkably the proportion of outpatients was very high (68%). The majority of patients (n = 42; 95.5%) were younger than 30 years.

All 44 *E. coli* strains were phenotypically confirmed as ESBL, PCR for *bla*_CTX-M_ and *bla*_CTX-M-1_ cluster was positive in all strains. Three exemplary isolates were chosen for sequencing of the full *bla*_CTX-M_ gene and sequence analysis revealed them as *bla*_CTX-M-15_. PCRs for IS*Ecp1* upstream and ORF 477 downstream of *bla*_CTX-M_ were positive in all strains. PCRs for TEM and OXA-1 beta lactamase genes were positive in 41 (93.1%) ESBL isolates. SHV could not be found in any isolate (Table 2). Also the presence of *aac(6′)-Ib-cr* could be demonstrated in 43 strains (97.7%) by PCR and subsequent restriction analysis. No significant difference regarding the frequency of *bla*_OXA-1_, *bla*_TEM_ and *aac(6′)-Ib-cr* between ESBLs from hospital A and Hospital B could be found. RAPD analysis of ESBL strains for clonal relatedness grouped the strains into seven clonal groups (A-G). Clone A was by far the most frequent clone and found in 65.9% of all studied strains. Compared to other clones it is more frequent in hospital B (81%) than in hospital A (57.1%). PCR results in relation to clonal type are presented in Table 2. The association of CTX-M-1 cluster genes to TEM, OXA-1 and *aac(6′)-Ib-cr* is stronger in Clone A ESBLs than in other clonal types. ESBL plasmid could be successfully transformed in 19 (43.2%) out of 44 ESBL *E. coli*. Co-resistance to gentamicin, kanamycin, tobramycin, doxycycline and trimethropim-sulfamethoxazole was observed in most transformants (Table 3).

**Table 2:** PCR results in relation to RAPD groups (n = 44)

**PCR tests**	**Clona A (n = 29)**	**Other clones (n = 15)**	**Total**
^bla^_*CTX-M-1*_	29(100%)	15(100%)	44(100%)
ISE*cp1*	29(100%)	15(100%)	44(100%)
ORF 477	29(100%)	15(100%)	44(100%)
^bla^_*SHV*_	0(0%)	0(0%)	0(0%)
^bla^_*TEM*_	26(89.7%)	15(100%)	41(93.1%)
^bla^_*OXA-1*_	28(96.5%)	13(85%)	41(93.1%)
*aac*(6)′-Ib-cr	28(96.5%)	15(100%)	3(97.7%)

**Table 3:** Characteristics of successful transformants

**Strains**	**Co-resistance**
10	CAZ, CTX, ATM, AMP, DO SXT, W
14	CAZ, CTX, ATM, AMP, TOB, CN, SXT, K
20	CAZ, CTX, ATM, AMP, DO, C, SXT, W
29	CAZ, CTX, ATM, AMP, K, TOB, CN, SXT
43	CAZ, CTX, ATM, AMP, SXT, DO, W, CN
48	CAZ, CTX, ATM, AMP, K, TOB, CN, DO
49	CAZ, CTX, ATM, AMP, K, TOB, CN DO
50	CAZ, CTX, ATM AMP, K, TOB, CN, DO, SXT, W.
60	CAZ, CTX, ATM, AMP, K, TOB, CN, DO
65	CAZ, CTX, ATM, AMP, K, TOB, CN, DO
73	CAZ, CTX, ATM, AMP, K, TOB, CN
74	CAZ, CTX, ATM, AMP, K, TOB, CN, DO, C, SXT, W
75	CAZ, CTX, ATM, AMP, K, TOB, CN
77	CAZ, CTX, ATM, AMP, K, TOB, CN,
78	CAZ, CTX, ATM, AMP, K, TOB, CN, DO
84	CAZ, CTX, ATM, AMP, DO, SXT, W
112	CAZ, CTX, ATM, AMP, SXT, C
131	CAZ, CTX, ATM, AMP, K, TOB, CN, DO
138	CAZ, CTX, ATM, AMP, K, TOB, CN

Key: *CAZ*-ceftazidime, *CTX*- cefotaxime, *ATM*- aztreonam, *AMP*- ampicillin, *TOB*- tobramycin, *SXT*- sulfametoxazole/trimethroprim, *W*- trimethroprim, *DO*- doxycycline, *CN*-gentamicin, *K*-kanamycin, *C*-chloramphenicol.

## Discussions

This study was carried out in two major university teaching hospitals in South-Eastern Nigeria where there is no record of investigation of molecular epidemiology of ESBL *E. coli*. Nosocomial bacterial infections constitute a substantial cause of morbidity and mortality in developing countries such as Nigeria. *E. coli* is known to be one of the major organisms causing nosocomial infections within the hospital and has also been implicated in community acquired ESBL [19,20]. The observation of ESBL producing *E. coli* strains in this study is very alarming and this could be attributed to the indiscriminate and widespread use of antibiotics, particularly beta lactam antibiotics that are sold over the counter in pharmacy shops without doctors’ prescription in Nigeria. This misuse of antibiotics might have contributed to the emergency of ESBL producing isolates. All 44 ESBL *E. coli* strains harbored ESBLs of the CTX-M-1 cluster. In three exemplary strains the CTX-M-15 ESBL could be confirmed which belongs to the CTX-M-1 cluster. These results further emphasize that this enzyme is now one of the most common CTX-M β- lactamases worldwide. In several countries CTX-M-15 is currently the most frequent ESBL in *E. coli* like in the UK, India, Spain, France, Latin America and Lebanon [20]. In Nigeria ESBL has been described in *Enterobacter* spp. from Western Nigeria several years ago [21] and CTX-M-15 ESBL in *K. pneumoniae* from the same region four years later [22] while ESBL was also reported in *E. coli* from South Eastern Nigeria this same year [23] but no description of CTX-M-15 ESBL from outpatients has been reported. Recently CTX-M-15 ESBL producing *E. coli* was reported from hospital patients in Osogbo in Western Nigeria [24]. CTX-M-15 has also been reported from other African countries like Egypt [25], Algeria [26], Tunisia [27], Tanzania [28], and Cameroon [29]. One of the most alarming findings of our study is that most ESBL isolates were detected in urine from young outpatients; urine was the source of 81.8% of ESBL strains, 68.2% of patients were of an age below 30 years. This strongly indicates that CTX-M-15 carrying *E. coli* strains can cause community-acquired urinary tract infections and that those ESBL strains are not only present in a hospital environment but also in the community population of this area of Nigeria. Similar observations have been made also in other parts of the world [5,6].

Typing of isolates via RAPD grouped the isolates into seven different clones. Seven strains could not be typed by this method and the majority of ESBL *E. coli* strains studied in this work belongs to a single clone as determined by RAPD. Of particular interest is that this clone A was detected in two hospitals located more that 70 km away from each other. One possible explanation is patient-to-patient transmission in both hospitals and introduction of clone A into another hospital by patient transfer. Hospital A serves as referral hospital for patients in hospital B with serious medical problems. A certain degree of inter-hospital spread was discovered because two patients with clone A ESBLs were known to have visited both hospitals during our study period. Thus, it is of high importance to apply specific infection control measures like hand disinfection in hospitals. Considering that also clone A ESBL isolates were found in young outpatients, another possible explanation might be that clone A occurs rather frequently in the population of this area. If so, it would be worth the effort to exclude a possible common source like drinking water or food products.

As observed worldwide for CTX-M-15 ESBL *E. coli* strains, we found a high association of CTX-M-1 cluster ESBLs with IS*Ecp1* upstream and ORF 477 downstream of the gene, with the presence of TEM and OXA-1 beta lactamases and with the detection of *aac(6′)-Ib-cr*. The latter gene mediates resistance to tobramycin and decreased susceptibility to ciprofloxacin [30]. Thus it is one of several factors responsible for multi-drug resistance also to non-beta lactam antibiotics in ESBL strains.

## Conclusions

For the first time ESBL *E. coli* strains could be demonstrated from Southeastern Nigeria with CTX-M-1 cluster enzymes present in all isolates. Most of the ESBL strains also carried TEM, OXA-1 and *aac(6′)-Ib-cr* and the majority of strains were isolated from outpatients below the age of 30. Based on our findings we suggest that antimicrobial resistance surveillance network be established in the two hospitals to monitor the trends and new types of resistance mechanism in Southeastern, Nigeria.

### Ethics

Ethical committee of the two hospitals approved the use of their hospitals for this study and patients gave consent for the collection of their samples.

## Competing interests

No interest to declare.

## Authors’ contributions

MKa and IRI planed this study. IRI performed most of the experiments. MKo, LM and SM performed parts of the experiments. MKa, FS and SG revised the manuscript. All authors read and approved the final manuscript.

## References

[B1] Medeiros AA Evolution and dissemination of β- lactamases accelerated by generations of β- lactam antibiotics Clin Infect Dis 1997 24 Suppl1 S19 S45 8994778 10.1093/clinids/24.supplement_1.s19

[B2] Bradford PA Extended-spectrum β- lactamases in the 21st century: Characterization, epidemiology, and detection of this important resistant threat Clin Microbiol Rev 2001 14 933 951 10.1128/CMR.14.4.933-951.2001 11585791 PMC89009

[B3] Amber RP Coulson AFW Frere JM Ghuysen JM Joris B Forsman M Levesque RC Tiraby G Waley SG A standard numbering scheme for the class A β- lactamases Biochem J 1991 276 269 272 2039479 10.1042/bj2760269 PMC1151176

[B4] Kassis-Chikhani V Vimont S Asselat K Trivalle C Minassian B Gautier V CTX-M- β- lactamase producing E. coli in long term care facilities, France Emerg Infect Dis 2004 10 1697 1698 10.3201/eid1009.030969 15503413 PMC3320274

[B5] Borer A Gilad J Menasche G Peied N Riesenberg K Schlaeffer F Extended-spectrum beta lactamase-producing Enterobacteriaceae strains in community-acquired bacteremia in Southern Israel Med Sci Monit 2002 8 CR44 CR47 11791130

[B6] Pitout JDD Normann P Laupland KB Poirel L Emergence of Enterobactericeae producing extended-spectrum beta-lactamases (ESBLs) in the community J Antimicrob Chemother 2005 56 52 59 10.1093/jac/dki166 15917288

[B7] Pitout JDD Gregson DB Church DL Elsayed S Laupland KB Community-wide outbreaks of clonally related CTX-M-14 β- lactamase-producing Escherichia coli strains in the Calgary health region J Clin Microbiol 2005 43 2844 2849 10.1128/JCM.43.6.2844-2849.2005 15956407 PMC1151948

[B8] Paterson DL Bonomo RA Extended-spectrum β- lactamases: a clinical update Clin Microbiol Rev 2005 18 657 686 10.1128/CMR.18.4.657-686.2005 16223952 PMC1265908

[B9] Poirel L Kamfer P Nordmann P Chromosomal-encoded Amber class A β- lactamases of Kluvera georgiana, a probable progenitor of a subgroup of CTX-M extended spectrum β- lactamases Antimicrob Agents Chemother 2002 46 4038 4040 10.1128/AAC.46.12.4038-4040.2002 12435721 PMC132763

[B10] Murray PR Manual of Clinical Microbiology 2002 7 Washington DC USA: American Society for Microbiology Press

[B11] Pineda FJ Lin LS Pensalau C Dinarov PA Testing the significance of micro-organism identification by mass spectrometry and protein database search Anal Chem 2000 72 3739 3744 10.1021/ac000130q 10959957

[B12] Pitout JDD Ashfaque H Hanson HD Phenotypic and Molecular detection of CTX-M-β-lactamses produced by Escherichia coli and Klebsiella spp J Clin Microbiol 2004 42 12 5715 5721 10.1128/JCM.42.12.5715-5721.2004 15583304 PMC535227

[B13] Schlesinger J Navon Venezia S, Chmelnistsky I, Hammer-Munz O, Leavitt A, Gold HS, Extended spectrum beta-lactamases among Enterobacter isolates Obtained in Tel Aviv, Israel, Antimicrob Agents Chemother 2005 49 3 1150 1156 10.1128/AAC.49.3.1150-1156.2005 PMC549242 15728917

[B14] Empel J Baraniak A Literacka E Mrowka A Fiett J Hryniewicz EW Beta Lactamase study group: Molecular survey of beta-lactamases conferring resistance to newer beta-lactams in enterobacteriaceae isolates from Polish hospitals Antimicrob Agents Chemother 2008 52 7 2449 2454 10.1128/AAC.00043-08 18458126 PMC2443902

[B15] Karisik E Ellington MJ Warren RE Livermore DM Woodford N Molecular characterization of plasmids encoding CTX-M-15 beta-lactamases from Escherichia coli strains in the United Kingdom J Antimicrob Chemother 2006 58 3 665 668 10.1093/jac/dkl309 16870648

[B16] Park CH Robicsek A Jacoby GA Sahm D Hooper HC Prevalence in the United States of aac(6′)-Ib-cr encoding a ciprofloxacin-modifying enzymes Antimicrob Agents Chemother 2006 50 3053 3055 10.1128/AAC.00915-06 PMC1635235 16954321

[B17] Eckert C Gautier V Arlet G DNA sequence analysis of the genetic environment of various bla CTX-M genes J Antimicrob Chemother 2006 57 1 14 23 16291869 10.1093/jac/dki398

[B18] Pacheco AB Guth BE Soares KC Nishimura L de Almeida DF Ferreira LC Random amplification of polymorphic DNA reveals serotype-specific clonal clusters among enterotoxigenic Escherichia coli from humans J Clin Microbiol 1997 35 6 1521 5) 9163473 10.1128/jcm.35.6.1521-1525.1997 PMC229778

[B19] Jacoby GA Han P Detection of extended-spectrum - lactamases in clinical isolates of Klebsiella pneumoniae and Escherichia coli J Clin Microbiol 1996 34 908 911 8815106 10.1128/jcm.34.4.908-911.1996 PMC228915

[B20] Woodford N Ward ME Kaufmann ME Turton J Fagan EJ James D Community and hospital spread of Escherichia coli producing CTX-M extended-spectrum β-lactamases in the UK J Antimicrob Chemother 2004 54 735 743 10.1093/jac/dkh424 15347638

[B21] Aibinu IE Ohaegbulam VC Adenipekun EA Ogunsola PT Odugbemi TO Mee BJ Extended spectrm beta lactamases in clinical isolates of Enterobacter species from Lagos, Nigeria J Clin Microbiol 2003 56 241 249 10.1128/JCM.41.5.2197-2200.2003 PMC154721 12734278

[B22] Soge OO Queenan AM Ojo KK Adeniyi BA Roberts MC CTX-M-15 extended spectrum beta lactamases from Nigeria Klebsiella pneumoniae J Antimicrob Chemother 2006 57 24 30 16319181 10.1093/jac/dki429

[B23] Iroha IR Adikwu MU Esimone CO Aibinu I Amadi ES Extended spectrum beta lactamase (ESBL) in E. coli isolated from a tertiary hospital in Enugu State, Nigeria Pak J Med Sci 2009 25 2 279 282

[B24] Olowe OA Grobbel M Buchter B Lubke-Becker A fruth A Wieler LU Detection of bla CTX-M-15 extended-spectrum β- lactamase genes in Escherichia coli from hospital patients in Nigeria Int J Antimicrob Agents 2010 35 200 209 10.1016/j.ijantimicag.2009.11.001 20022221

[B25] Khalaf NG Eletreby ME Hanson ND Characterization of CTX-M ESBLs in Enterobacter cloacae, Escherichia coli and Klebsiella pneumoniae from Cairo, Egypt BMC Infect Dis 2009 9 84 1 5 19497111 10.1186/1471-2334-9-84 PMC2701952

[B26] Ramdani-Bouguessa N Mendorica N Levtao J Ferreira E Tazir M CTX-M-3 and CTX-M-15 extended spectrum β- lactamases in isolates of Escherichia coli from a hospital in Algiers, Algeria J Clin Microbio 2006 44 12 4584 4586 10.1128/JCM.01445-06 PMC1698418 16988017

[B27] Boualiegue-Godet O Bensalem Y Fabre L Demartin M Grimont PAD Mzoughi R Nosocomial outbreak caused by Salmonella enteric serotype producing CTX-M-27 extended-spectrum β-lactamase in a neonatal unit in Sousse, Tunisia J Clin Microbiol 2005 43 1843 1849 10.1128/JCM.43.4.1843-1845.2005 15750057 PMC1081247

[B28] Blomberg B Jureen R Manji KP Tamin BS Mwakagile DSM High rate of cases of pediatric septicemia caused by Gram-negative bacteria with extended spectrum β-lactamases in Dar es Salaam, Tanzania J Clin Microbiol 2005 43 745 749 10.1128/JCM.43.2.745-749.2005 15695674 PMC548071

[B29] Gangoue-Pieboji J Miriagou V Vourli S Tzelepi E Ngassam P Tzouvelekis LS Emergence of CTX-M-15-producing enterobacteria in Cameroon and characterization of a bla CTX-M-15- carrying element Antimicrob Agents Chemother 2005 49 1 441 443 10.1128/AAC.49.1.441-443.2005 15616331 PMC538870

[B30] Strahilevitz J Jacoby GA Hooper DC Robicsek A Plasmid-mediated quinolone resistance: a multifaceted threat Clin Microbio Rev 2009 22 4 664 689 10.1128/CMR.00016-09 PMC2772364 19822894

